# Adenocarcinoma of the ascending colon in a 31-year-old pregnant woman: A case report

**DOI:** 10.1097/MD.0000000000013707

**Published:** 2018-12-21

**Authors:** Youzheng Xu, Beihua Kong, Keng Shen

**Affiliations:** a Department of Obstetrics and Gynecology, Peking Union Medical College Hospital, Chinese Academy of Medical Sciences and Peking Union Medical College, Eastern District, Beijing; b Department of Obstetrics and Gynecology, Qilu Hospital, Shandong University, Jinan, China.

**Keywords:** adenocarcinoma of the ascending colon, colorectal cancer, diagnosis, pregnancy, treatment

## Abstract

**Rationale:**

Colorectal cancer (CRC) is the 2nd most common type of cancer in females and the 3rd in males, worldwide. It occurs rarely during pregnancy and is often associated with poor prognosis, due to the unspecific manifestations until advanced stage. Majority of CRC are localized in the rectum (63%) and the sigmoid colon (20%) during pregnancy.

**Patient concerns:**

In thisstudy, we report the case of a pregnant woman who was diagnosed with adenocarcinoma of the ascending colon at her 33rd gestational week. She was referred to our department from local hospital with low fever and right-sided flank pain, which had lasted for nearly half a year and severely aggravated for 5 days. Previous prenatal examinations contributed the pain to kidney stones or uterine contractions.

**Diagnoses:**

After a caesarean section and tumor resection of a mass at the hepatic flexure of colon, tumor histology of frozen section confirmed the diagnosis of ulcerative adenocarcinoma of the ascending colon with a diameter of 10 cm. Final pathologic evaluation showed a grade 1 adenocarcinoma with negative lymph nodes (16/0), R0 resection, pT4b pN0 M0 and Dukes B stage.

**Interventions:**

A healthy female infant was delivered by caesarean section, right after which a right hemicolectomy and ileostomy was performed. Pathology examination proved an early stage adenocarcinoma with no lymphatic metastasis. Patient received chemotherapy with folinic acid, fluorouracil, and oxaliplatin (FOLFOX) after recovery smoothly and got discharged 1 month after surgery.

**Outcomes:**

Patient showed no relapse or progression during the follow-up time of 2 years after operation and chemotherapy.

**Lessons:**

Rare occurrence of CRC during pregnancy and limited experience concerning its diagnosis and treatment bring obstacle to both patients and physicians. Symptoms as constipation and abdominal pain must be inspected carefully. With a perfect coordination between different disciplines, CRC with pregnancy can be ideally treated with better prognosis.

## 1 Introduction

Colorectal cancer (CRC) is the 2nd most common type of cancer in females and the 3rd in males worldwide,^[1]^ which rarely occurs during pregnancy. Reported incidence in pregnant women is 0.002%^[2]^ and the majority of tumors were localized in the rectum (63%) and the sigmoid colon (20%), which is contrary to the general population, and Dukes stage at presentation was B or higher in all patients.^[3]^

Typical symptoms of CRC include nausea, vomiting, abdominal pain, and altered bowel movements. All the symptoms above can be misdiagnosed as common symptoms of normal pregnancy. Considering the potential risks of further diagnostic tests to the fetus such as colonoscopy or computed tomography (CT), physicians and patients tend to ignore the underlying risks. Perhaps due to the immunotolerance which accompanies pregnancy, colon obstruction, perforation, and metastatic spread seem to occur more frequently in these patients, which leads to the advanced development of malignancy and poorer prognosis.^[4]^

Because of the rare occurrence of CRC during pregnancy, there is limited experience concerning its diagnosis and treatment, which requires more knowledge and clinical experience to support both patients and physicians. To the best of our knowledge, we present the rare case of adenocarcinoma of the ascending colon during pregnancy in this study. The patient was successfully treated surgically and a healthy female infant was delivered by caesarean section.

## 2 Case report

A 31-year-old woman (gravida 4, para 1) was referred to our department from local hospital at 33rd weeks gestation with low fever and right-sided flank pain, which had lasted for nearly half a year and severely aggravated for 5 days. As the patient recalled, previous prenatal examinations in local hospital contributed the pain to kidney stones or uterine contractions without any further inspection. After local outpatient treatment with antibiotics, progesterone and Nonsteroidal Antiinflammatory Drugs (NSAIDs), she was admitted to our department as the pain aggravated. She denied any vomiting, hematochezia, or difficulty with urination.

**Figure 1:**
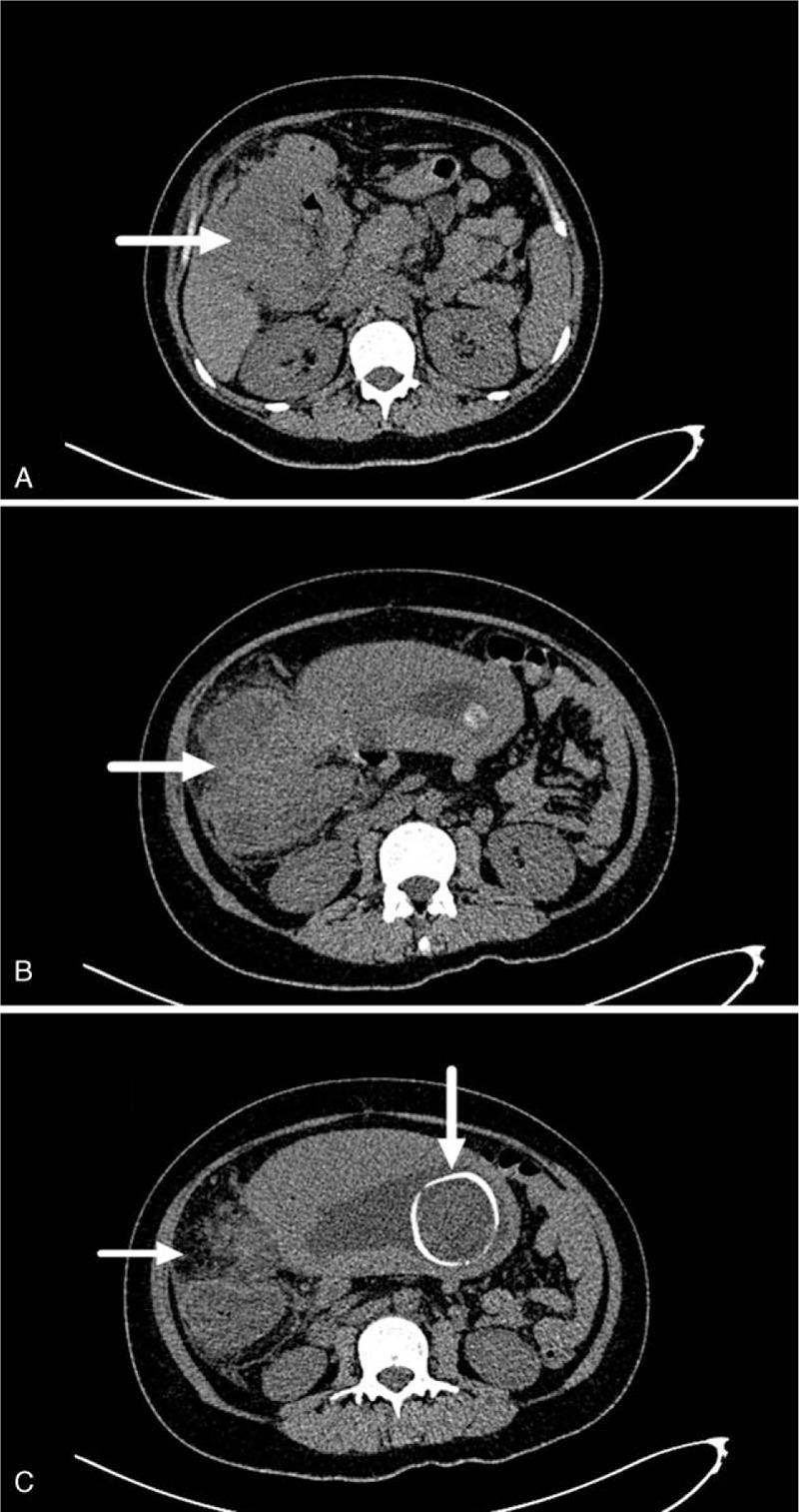
Computed tomography abdomen images. (A) Incrassation of the ascending colon wall and exudative change around it. (B) Tumor extended to ileocecus of the colon. (C) The skull of the fetus can be seen with similar diameter to the tumor.

Moderate iron deficiency anemia, occasional dyspepsia, and diarrhea were present during the whole gestation period. At the time of presentation, patient had no family history of gynecologic or CRC. Physical examination revealed right-sided abdominal pain on palpation and normal bowel sounds. Vital signs are normal. Body mass index 24.8. Obstetric examination showed no abnormalities. Initial laboratory results included a mildly elevated white cell count and hemoglobin 7.6 g/dL and a mean corpuscular hemoglobin 26.7 pg. Her serum potassium was 4.0 mmol/L. Liver function tests showed the serum albumin was 20 g/L. Urinalysis and routine excrement examination remained normal with no occult blood. Tumor markers serum carcinoembryonic antigen (CEA) was elevated to 70.68 ng/mL. Abdominal ultrasound showed a large heterogeneous cystic mass located below the hepatic flexure of colon. Considering the extremely low risk of radiation teratogenicity in late pregnancy, an abdominal CT scan without contrast was obtained, which revealed incrassation of the ascending colon wall and exudative change around it (Fig. 1). Multiple pathologically enlarged abdominal lymph nodes were observed. No colonoscopy was performed considering the site of the lesion and the possibility to induce uterus contraction of premature delivery.

After a detailed discussion in a multidisciplinary medical team, a planned delivery by caesarean section and tumor resection during the same operative procedure was performed, following the induced fetal lung maturation by dexamethasone. Intraoperatively, a palpable tumor mass at the hepatic flexure of colon with a diameter of 15 cm was identified, with the right lobe of the liver and perirenal adipose tissue involved. Mucopurulent discharge could be found through the rupture on the necrotic mass. Further abdominal exploration revealed paracolic lymph nodes, intermediate lymph nodes, and middle lymph nodes pathologically enlarged, with none metastatic lesions on pancreas or duodenum. Both adnexa and the rest of the peritoneal cavity also appeared normal. A 1550-g live female infant with an Apgar score 10-10-10 was delivered at 33rd of gestation. A right hemicolectomy and ileostomy were performed right after the cesarean section. Tumor histology of frozen section confirmed the diagnosis of ulcerative adenocarcinoma of the ascending colon with a diameter of 10 cm. Final pathologic evaluation showed a grade-1 adenocarcinoma with negative lymph nodes (16/0), R0 resection, pT4b pN0 M0, and Dukes B stage. The postoperative course was uneventful while patient received chemotherapy with folinic acid, fluorouracil, and oxaliplatin (FOLFOX) after recovery smoothly and got discharged 1 month after surgery. Patient showed no relapse or progression during the follow-up time of 2 years.

Written informed consent was obtained from the patient for publication of the present case report and any relevant images.

## 3 Discussion

The most common malignancies complicated with pregnancy are breast cancer, cervical cancer, lymphoma, ovarian cancer, and melanoma,^[5–10]^ which possess a peak incidence during the woman's reproductive period. CRC presents in only 1 in 13,000 pregnancies.^[3]^ There has been much controversy on the role of estrogens and progestogens in malignancy during pregnancy. Neoplastic cell cultures in vitro can be stimulated by elevated estrogens and progestogens. On the contrary, recent report showed the incidence of CRC is not influenced by the number of gestations or contraceptive use. Experimental data reported a lack of estrogen/progesterone receptors in CRC.^[11]^ However, a prospective study showed that parity may reduce the risk of colon cancer among women as a result of modifications of hormone profiles.^[12]^ Possible mechanisms include hormonal effects on bile acid metabolism^[12,13]^ and immunologic influences of ABO-incompatible fetal antigens.^[14]^

Diagnosis of CRC during pregnancy is quite challenging and often delayed because the symptoms (e.g., constipation, abdominal pain, change in bowel habits, nausea, and vomiting) are unspecific and usually attributed to general manifestations of pregnancy without further diagnostic tests considering the potential fetal risks. Therefore, pregnant women are diagnosed with locally advanced or metastatic CRC more often than general population and have a substantially poorer prognosis.^[15]^ During pregnancy, CEA levels are inevitable increased but within normal range. But elevated CEA value before surgery can be used as a prognostic factor for recurrence and disseminated disease.^[16]^ Colonoscopy is the most definite diagnostic tool which can provide precise biopsy and pathologic examination in general patients with CRC. However, during pregnancy colonoscopy possess the potential risks of uterine pressure, placental abruption, and fetal injury. Abdominal ultrasound is a rather safe diagnostic method compared to CT, which is the standard imaging technique used for determining CRC. Magnetic resonance imaging (MRI) is the imaging tool of choice as abdominal CT is contraindicated in pregnancy for its radioactive teratogenic and carcinogenic effects on the fetus.^[17]^ Unenhanced MRI is preferred because safety of contrast agents during pregnancy has not been proved.^[18]^ In this case, a plain abdominal CT is obtained instead of MRI because the contraindications during this gestational period is relatively negligible. Meanwhile a CT scan is more detailed on the depth of carcinoma tissue infiltration, lymph node metastasis, and birth canal obstruction. There has been researches showing that CRC pose no threat to the fetus.^[19]^ It is reported that 25 patients delivered normal infants among 32 cases of pregnancy complicated by colon carcinoma.^[20]^ Metastasis to the placenta or embryo is extremely rare as only 1 case has been reported till now, and the infant proved to be normal in the follow-up of 8 months.

To optimally treat the mother and minimize the side effect on the fetus, treatment of CRC during pregnancy must be considered thoroughly from many aspects, including the gestational stage, elective or emergency presentation, the progression of the disease, and the wishes of the patient. Gestational weeks ≤20 weeks: surgical resection is recommended right after diagnosis to minimize the progression of malignancy. The incidence of premature delivery and low birth weight followed operation is reported to be double that of the general normal pregnancies.^[4]^ Artificial abortion should be considered on personal wishes. Adjuvant chemotherapy or radiotherapy depends on the stage of the cancer. Three cases have been reported of chemotherapy use during pregnancy without any observable adverse effect on the fetus.^[21–23]^ Gestational weeks ≥20 weeks: the operation is recommended to be delayed until a viable fetus is delivered (28–30 weeks), with certain drugs administered to induce fetal lung maturation. Choice between vaginal delivery and caesarean section has been controversial among authors. Resection of tumor should be performed usually 1 to 2 weeks after vaginal delivery when the involution of the uterus and resolution of vascular congestion of pelvic structure completes.^[24]^ In this case, we chose cesarean section to avoid high pressure or trauma to the tumor during vaginal delivery and reduced the harm to the patient by performing tumor resection at the same setting.

Incidence of metastasis to ovaries in CRC is reported to be 3% to 8% among general population but 25% during pregnancy.^[25,26]^ Visual inspection and biopsy of ovaries are highly recommended in the operation, but ovariectomy should not be performed unless metastasis is detected or hysterectomy is unavoidable. Chemotherapy is recommended in patients of Dukes C stage or incomplete excision of the tumor. The 5-fluorouracil-based chemotherapy is relatively safe during the 2nd and 3rd trimester of gestation with lower risk of abortion or malformation (2–3%) compared to the first trimester (15–25%).^[27]^ However, growth retardation and premature labor can still be found, which leaves the decision-making process to the patient herself about whether to apply chemotherapy. Radiotherapy is abandoned during pregnancy and not recommended with the patient willing to bear again since colon cancer is not sensitive to radiotherapy and ovarian function can be devastated.

The prognosis of CRC is about the same at each stage for both pregnant and general patients. However, gravid women are usually delayed in diagnosis, which leads to more advanced and malignant disease and poorer prognosis. In a research of 39 cases of CRC during pregnancy in United States, 16 were diagnosed at Dukes B stage, 17 at Dukes C, and 6 at Dukes C.^[3]^ In conclusion, early diagnosis, active treatment and more attention to unspecific symptoms from clinicians during pregnancy is the key to reverse this unfavorable situation.

## Author contributions

**Investigation:** Youzheng Xu.

**Supervision:** Beihua Kong, Keng Shen.
